# Sinus pilonidalis—elastic ligature as an optimal outpatient treatment

**DOI:** 10.1007/s00104-023-02014-5

**Published:** 2023-12-14

**Authors:** Ľudovít Danihel, Marián Černý, Matúš Rajčok, Kristína Mosná, Jihad Bou Ezzeddine, Ivor Dropco, Milan Schnorrer

**Affiliations:** 1 3rd Surgical Clinic, Faculty of Medicine, Comenius University in Bratislava, Bratislava, Slovakia; 2 Klinik für Allgemein‑, Viszeral‑, Thorax‑, Adipositas‑, Gefäß- und Kinderchirurgie, Passau, Germany; 3 Institute of Pathological Anatomy, Faculty of Medicine, Comenius University in Bratislava, Bratislava, Slovakia; 4 2nd Geriatric Department, Faculty of Medicine, Comenius University in Bratislava, Bratislava, Slovakia; 5 Klinik und Poliklinik für Chirurgie, Universitätsklinikum Regensburg, Regensburg, Germany

**Keywords:** Pilonidal sinus, Ligation, Excochleation, Local anesthesia, Sacrococcygeal region, Pilonidalsinus, Ligatur, Exkochleation, Lokalanästhesie, Sakrokokzygealregion

## Abstract

**Background:**

The incidence of pilonidal sinus shows a steadily rising tendency, especially in the patient age group of up to 40 years. Treatment of this condition is often protracted involving lengthy sick leave and an increased risk of recurrence. The optimal treatment of pilonidal sinus remains open to debate, but it should focus on decreasing the length of hospitalization, promoting a rapid return to daily life, maintaining low pain levels, and keeping costs at a minimum.

**Materials and methods:**

In our study conducted between 2017 and 2021, we focused on treatment of pilonidal sinus. We performed 50 elastic ligature procedures with a median observation time of 30 months. The patients were divided into three groups according to the characteristics of pilonidal sinus: (1) acute primary abscess; (2) acute recurrent abscess; and (3) chronic fistula.

**Results:**

Out of a total of 50 patients with a subsequent 30-month follow-up, we observed complete recovery in 47 patients and recurrence in three patients. Return to work was possible immediately after the operation, with an average total treatment time of 1 month for complete healing of the defect.

**Conclusion:**

The current results suggest that the technique of elastic ligature is a desirable solution for pilonidal sinus, because of the initial low costs, no need for hospitalization, and good patient tolerance.

## Abstract (de)

**Hintergrund:**

Die Inzidenz des Pilonidalsinus weist eine stetig steigende Tendenz auf, insbesondere in der Patientengruppe mit einem Alter bis zu 40 Jahren. Die Behandlung dieser Erkrankung ist oft langwierig – mit einer langen Arbeitsunfähigkeit und einem erhöhten Rezidivrisiko. Dabei bleibt die optimale Therapie des Pilonidalsinus umstritten, aber sie sollte sich auf die Verminderung der Dauer des Krankenhausaufenthalts, die Förderung einer schnellen Rückkehr in den Alltag, die Aufrechterhaltung niedriger Schmerzlevel und die Begrenzung der Kosten auf ein Minimum konzentrieren.

**Material und Methoden:**

In der vorliegenden Studie, die zwischen 2017 und 2021 durchgeführt wurde, lag der Fokus auf der Behandlung des Pilonidalsinus. Dabei erfolgten 50 Eingriffe mit elastischer Ligatur und einer medianen Nachbeobachtungsdauer von 30 Monaten. Die Patienten wurden nach den Charakteristika des jeweiligen Pilonidalsinus in 3 Gruppen unterteilt: (1) akuter primärer Abszess; (2) akuter rezidivierender Abszess und (3) chronische Fistel.

**Ergebnisse:**

Von insgesamt 50 Patienten mit einem anschließenden Follow-up von 30 Monaten wurde bei 47 Patienten eine vollständige Heilung und bei 3 Patienten ein Rezidiv festgestellt. Die Wiederaufnahme der Arbeit war unmittelbar nach der Operation möglich, dabei betrug die durchschnittliche Gesamttherapiedauer einen Monat bis zur vollständigen Heilung des Defekts.

**Schlussfolgerung:**

Die aktuellen Ergebnisse liefern Hinweise darauf, dass die Technik der elastischen Ligatur eine wünschenswerte Lösung zur Therapie des Pilonidalsinus darstellt, da die initialen Kosten niedrig sind, ein Krankenhausaufenthalt nicht notwendig ist und eine gute Verträglichkeit durch die Behandelten besteht.

## Introduction

Pilonidal sinus can be defined as a chronic inflammation of the subcutaneous tissue in the sacrococcygeal region in the area of the intergluteal cleft. The diagnosis was first described by O.H. Mayo as early as 1833 [1]. Despite a large number of publications describing different surgical tactics in the treatment of this entity, the optimal therapy still remains open to debate. Commonly used surgical procedures, such as the Z‑flap, Karydakis procedure, V‑Y plasty technique, plastic procedure with Bascom flap, and Limberg flap, result in excision of the pilonidal sinus with open healing or with a primary closure. The goal of these extensive procedures followed by reconstructive plastic surgery is the complete removal of all diseased tissues with a primary closure of the wound distant from the central line [2, 3].

Disadvantages of the aforementioned surgical techniques include the extent of the procedure, modification of the anatomy of the intergluteal cleft, protracted recovery period, risk of wound dehiscence in the case of plastic procedures, and no significant decrease in the risk of recurrence [4]. Minimally invasive techniques, such as pit-picking or the use of fibrin glue in simple cases of pilonidal sinuses, showed satisfactory results in terms of shorter hospitalization and faster return to active life [5, 6]. The latest technique used for the treatment is represented by a radial laser probe (Fistula-tract Laser Closure [FiLaC]; [7]). The question that is raised in this setting is whether using expensive techniques is reasonable [8]. On the other side is the LOCULA procedure—laying open and curettage of the pilonidal sinus under local anesthesia. It is a very good option for the treatment of pilonidal sinus, and has comparable results to our technique with elastic ligature; however, LOCULA is more invasive to the tissues. The incidence of pilonidal sinus disease is 26/100,000 population, with a twofold higher occurrence in male patients [9]. It is commonly referred to as “jeep driver’s disease.”

An adequate alternative to common plastic procedures could be represented by the treatment of pilonidal sinus with elastic ligature, as it involves minimal costs and at the same time has been shown to offer greater patient tolerance with a rapid return to daily activities.

## Materials and methods

Between 2017 and 2021, we performed 50 elastic ligature procedures with a median follow-up time of 30 months. The study included 34 male patients and 16 female patients, with an average age of 27.5 years. It was found that 15 patients had signs of chronic pilonidal sinus disease and 35 patients presented with acute pain and inflammation of the intergluteal cleft. The patients were divided into three groups according to the characteristics of pilonidal sinus: 20 patients with acute primary abscess in group 1; 15 patients with acute recurrent abscess in group 2; and 15 patients with chronic fistula in group 3. The average length of clinical symptomatology in patients with recurrent abscess and chronic fistula was 4.7 years until the procedure was performed (Table 1).

**Table 1:** Patient characteristics and postoperative outcomes

*Number of patients*	50		
Male	34		
Female	16		
*Average age*	27.5 (19–36)		
*Type of sinus pilonidalis*	Acute primary abscess	Acute recurrent abscess	Chronic fistula
	20	15	15
*Mean operative time (min)*	21	26	34
*Average fistulotomy (days)*	12	21	26
*Return to work (days)*	Immediately	Immediately	Immediately
*Average return to sport (days)*	28	40	31
*Postoperative outcomes at 30-month follow-up*			
Healed	20	14	13
Recurrence	0	1	2
*Visual Analog Scale score (from operation day)*			
1–3 (mild pain)	18	11	11
4–6 (moderate pain)	2	4	4
7–10 (severe pain)	0	0	0
*Level of patient satisfaction after 30 months*			
1: Very satisfied	18	11	12
2: Satisfied	1	1	3
3: Neither satisfied or dissatisfied	1	2	0
4: Dissatisfied	0	1	0
5: Very dissatisfied	0	0	0

### Treatment procedure

**Fig. 1:**
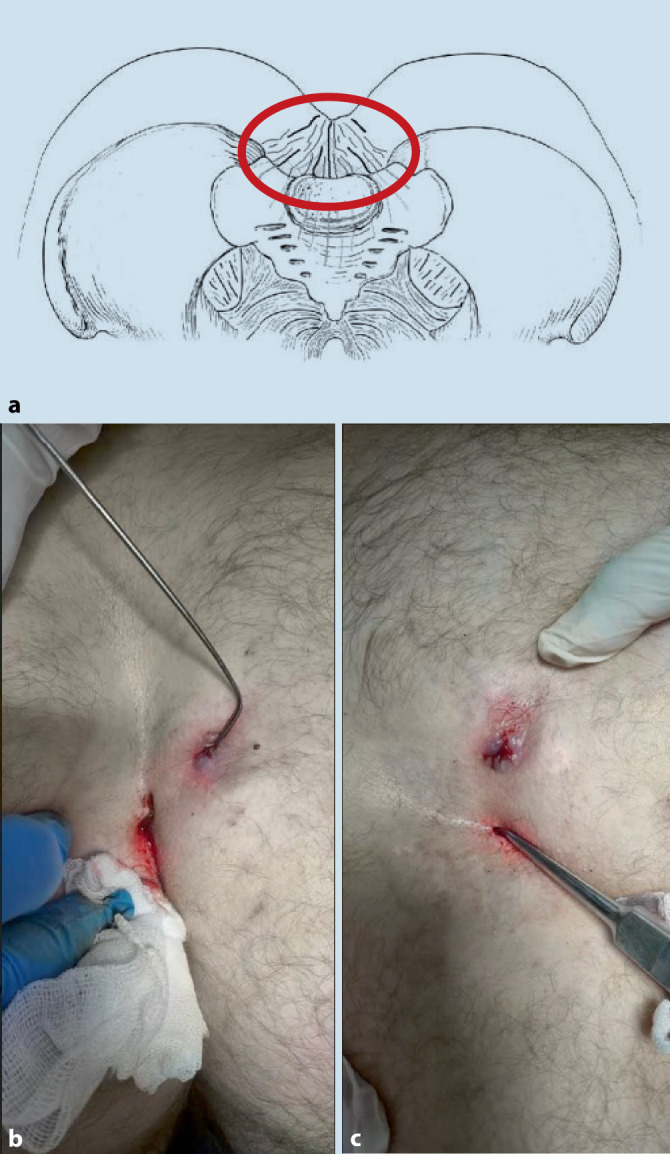
**a** Anatomic definition of the disease; **b** identification of the whole sinus; **c** excochleation of the sinus

**Fig. 2:**
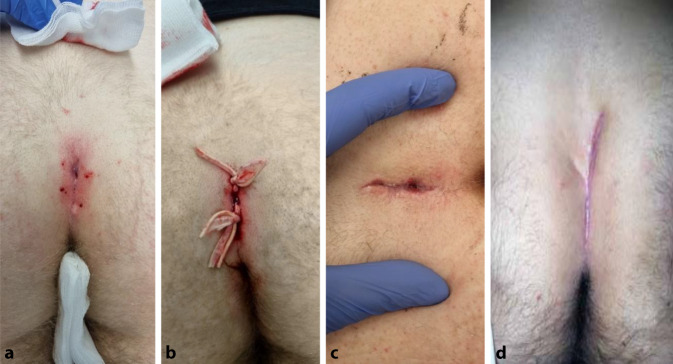
Operation steps: **a** Local anesthesia; **b** after excochleation and identification of the whole cavity, a ligature is placed; **c** almost healed defect (own material); **d** after complete healing [19]

The treatment procedure was performed with patients under local anesthesia using 1% Mesocain. However, Marcaine had to be used in three cases because of Mesocain intolerance. After depilation of the intergluteal cleft, the fistulas of the pilonidal sinus were identified. In the group of patients with an acute abscess, a primary incision was performed with abscess drainage followed by excochleation of the walls of the abscess. After primary removal of the inflammatory focus and initial signs of granulation, we indicated ligature treatment. Consequently, probing of the sinus and fistula was carried out in order to identify the entire course of the fistula, including the distant communicating focuses. During this time, contra-incisions were made, through which the elastic ligature was later threaded (Fig. 1). Both thorough excochleation of the entire cavity and thorough rinsing with an antimicrobial solution are extremely important. If the skin bridge between the incision and the contra-incision is too long, it can be divided, and thus more elastic ligatures can be added (Fig. 2). The average procedure time is 30 min.

Postoperative care is carried out at home with a careful daily rinse of the wound, which is followed by topical application of a remedy promoting the formation of granulation tissue. Outpatient appointments are scheduled on a weekly basis with the aim of fastening the ligature and applying the antimicrobial solution as needed. The interval from the falling off of the elastic ligature to the formation of a solid narrow is usually 1 month. It is important to prevent premature closure of the tissue over the unhealed cavity. The correct placement of the covering is one of the key factors required for successful healing of the defect.

## Results

The average duration of fistulotomy was 20 days in our study, with complete healing of the fistula lasting 29 days. The average period of postoperative follow-up was 30 months. During this time, we observed two cases of recurrence with a deep defect at the edge of the pilonidal sinus and one case with protracted healing of 45 days. According to the Visual Analog Scale (VAS) score, up to 86% of patients experienced no pain or only mild pain in the postoperative period, and only 14% of patients showed moderate pain levels. We observed great patient satisfaction with this method in 89% of cases, which we consider significant in view of the patient sample size.

## Discussion

The optimal treatment of pilonidal sinus remains open to debate. However, decreasing the length of hospitalization, a faster return to daily life, low pain levels, good long-term results, and the cost of the treatment should all be taken into consideration.

In the past, there was a preference for excision of the pilonidal sinus with open healing or plastic surgery techniques. The disadvantage of these treatments lies mainly in the change in the anatomy of the intergluteal cleft, which could cause discomfort especially while sitting, as well as a longer convalescence period. In the case of plastic techniques, there are currently recommendations against midline sutures and instead the use of off-midline sutures is recommended, which is associated with a lower rate of dehiscence [10]. These treatments of pilonidal sinus are associated with a high rate of complications and a long and painful postoperative period requiring daily wound care, with a decrease in the patients’ quality of life. Overtreatment with the use of excessively radical excisions and skin flaps in cases of simple pilonidal sinuses increases the risk of complications and the length of hospitalization and decreases the positive cosmetic effect [11]. Other therapeutic possibilities include negative-pressure treatment. For example, Giordano et al. (2021) published their results with this treatment option: They included 13 patients, and achieved complete healing of the defect in 11 patients [12]. Milone (2014) published the results of endoscopic treatment of the pilonidal sinus [13]. Cahis (2021) described the exact mechanisms of this treatment option, with a healing period of 15–30 days [14]. The implementation of minimally invasive techniques is debated, especially since the expenses of the treatment and the recurrence of the disease are not significantly lower [15].

The tendency to use expensive devices should be avoided, especially when they do not provide any extra advantage. A meta-analysis by Garg et al. (2021) reported a recurrence rate of 4.47% for the LOCULA procedure; up to 11.9% in the patient group with excision with open healing (ExOH); up to 7.1% in the patient group with marsupialization (ExMars); up to 20% in the case of excision with midline closure (ExMC); and up to 11% in the case of excision with off midline closure with a flap (ExOMC‑F; [3, 16]). The pooled recurrence rate in this meta-analysis was 4.47%, and 84 days for return to work [17].

The LOCULA procedure is not merely an open drainage of the pilonidal sinus. During this procedure, deroofing (laying open) is performed, and the overhanging margins are partially trimmed to create a saucer-shaped wound. This helps prevent adherence of wound edges, thus promoting healing by secondary intention [18]. Return to work after LOCULA was possible after 3.6 ± 2.9 days, with a healing time of 43.8 ± 7.4 days, in contrast to the method using ligatures, where immediate return to work was possible with a total treatment duration of up to 28 days (± 5 days; [18]).

Our work unequivocally proved that the use of elastic ligatures is an optimal variant in the treatment of pilonidal sinus disease. The benefits include the out-patient treatment, minimal pain, great patient tolerability, and low treatment costs. We found out that return to work was possible immediately after the operation, which is an important difference to the other techniques.

Our results are significant in comparison to the results of Garg’s meta-analysis. The aim of the LOCULA procedure is causal treatment with definite removal of pathologically changed tissue [18]. The keystone of this technique is the slow fistulotomy performed with the placement of elastic ligatures, while the whole course of the sinus must be included. The principle is similar to the treatment of anal fistula. Thanks to similar pathomechanisms of these diseases, the use of elastic ligatures is also appropriate for pilonidal sinus [20, 21]. The treatment of the entire area of the defect decreases the risk of recurrence of pilonidal sinus. The advantage of this treatment is a very good control of the granulation due to the slow cutting of the skin by means of ligation, and thus the reduced risk of premature closure of the cavities, which could be a reason for recurrence. It is very important to pay attention both to depilation of the affected area throughout the treatment period and to permanent depilation after healing of the defect.

## Conclusion

The incidence of pilonidal sinus shows a steadily rising tendency, especially in the patient age group of up to 40 years. The treatment of this condition is often protracted with lengthy sick leave and an increased risk of recurrence. Several types of therapeutic options are known. Recently, minimally invasive procedures have come to the fore, but they are very expensive. The tendency to use expensive gadgets should be avoided especially when they do not provide any extra advantage. Thus, it is very important to promote cheaper methods of treatment that have a comparable risk of recurrence and very good patient tolerance. The method described in this article fulfills these criteria in every respect.

## Data Availability

https://www.solen.sk/storage/file/article/f5e87bf463022e9dca86fc97be0a9bd4.pdf.

## Declarations

The statement of the ethics committee was not required for the study as per the rules in Slovakia.
